# Neuromagnetic Indication of Dysfunctional Emotion Regulation in Affective Disorders

**DOI:** 10.1155/2012/156529

**Published:** 2012-05-10

**Authors:** Christian Pietrek, Tzvetan Popov, Astrid Steffen, Gregory A. Miller, Brigitte Rockstroh

**Affiliations:** ^1^Department of Psychology, Zukunftskolleg, University of Konstanz, 78457 Konstanz, Germany; ^2^Department of Psychology, University of Delaware, Newark, 19716 DE, USA

## Abstract

Dysfunctional emotion regulation is often reported in affective disorders, but it is unclear whether this dysfunction concerns initial processing of emotional input or regulation of resulting emotion. The present study addressed these aspects in 27 depressive and 15 borderline personality disorder patients and 28 healthy controls who were instructed to either passively view unpleasant and neutral pictures or downregulate emotional responses by reappraisal, while neuromagnetic brain activity was measured. All three groups showed more early response to unpleasant than to neutral pictures, whereas patients failed to show subsequent activity suppression under instructions to down-regulate. This deficient emotion regulation was evident primarily in those subjects reporting high childhood adversity. Results support intact emotional input processing but impaired emotion regulation in affective disorders and indicate a moderating influence of early life stress.

## 1. Introduction

Impaired emotion regulation is often discussed as characteristic of disorders of affect. It is reported for major depressive disorder (MDD, [1–4]) and has been described as a core feature in borderline personality disorder (BPD, [5–8]). Dysfunctional emotion regulation could result from impaired initial processing of emotional input or from impaired regulation of physiological and behavioral aspects of resulting emotion. A widely cited model [9, 10] distinguishes perceptual input-oriented processes of monitoring, appraisal, or evaluation of an emotional stimulus and response- or output-oriented regulation processes that may include cognitive reappraisal or response suppression. Similarly, a prominent earlier model [11] centrally distinguished stimulus and response aspects of emotional processing. Research on neural mechanisms associated with this distinction has related input-oriented processes to amygdala and anterior cingulated gyrus (ACC, [10, 12]) and output-oriented regulation processes to ventromedial prefrontal cortex (VMPFC; [10, 13–17]). The interplay of these processes seems crucial for efficient regulation [9, 11].

Emotion dysregulation has been inferred from hemodynamic neuroimaging findings of reduced ventromedial frontal activity during reappraisal, affect discrimination, and emotional Stroop tasks in patients with MDD [1–3] and from augmented limbic activity and reduced orbitofrontal activity in patients with BPD [7]. Moreover, a limbic-prefrontal activity pattern under downregulation instructions opposite to the one characteristic of healthy subjects [2, 18] suggests that reappraisal, regulation, and their interplay are dysfunctional in affective disorders.

Hemodynamic neuroimaging results may not be sufficient to elucidate the temporal dynamics of emotion regulation, that is, how this interplay unfolds across time. Electro-(EEG) and magnetoencephalography (MEG) complement hemodynamic neuroimaging evidence of these dynamics with better temporal resolution. In healthy subjects, Moser and colleagues [19] described the time course of event-related potential (ERP) components based on sparse-array EEG when subjects were asked to implement cognitive reappraisal strategies for emotion regulation. Modulation of the late positive potential (LPP, some 400–700 ms after picture onset) by instruction to down-regulate the processing of unpleasant pictures (relative to passive viewing) indicated a temporal sequence of appraisal and regulation (see also [19–22] for modulation under instruction to suppress the emotional response to pleasant pictures). The present MEG study employed a design adapted from Moser et al. [19] to examine cortical activity during cognitive reappraisal in patients with MDD and BPD as well as psychiatrically healthy subjects. Examining the temporal sequence of processes in patients with affective disorders should help to identify mechanisms of this deficient interplay of processes, such as the extent to which they are serial or concurrent.

An additional motivation of the present study was to identify a potential impact of childhood adversity on emotion regulation. Various phenomena have been discussed as contributing to dysfunctional emotion regulation in affective disorders, among them childhood adversity. Adverse experiences early in life are believed to modify emotion processing throughout life via impact on stress reactivity [23] and on the development of neural systems involved in complex cognitive and affective functions [24]. Studying neuromagnetic activity in a picture-viewing task, we found less evoked brain activity to picture onset in patients with MDD or BPD, with higher childhood stress load associated with smaller responses, but an intact arousal effect [25]. The arousal effect has been interpreted as a robust, low-level, essentially automatic aspect of emotion processing indicating the tagging of cues as relevant [26]. Thus, this study provided evidence of intact automatic emotion processing in such individuals despite childhood adversity and despite the generally smaller brain activation. A next step in understanding emotion processing in such individuals would be assessing their performance in a task providing an opportunity for more active processing of emotional stimuli. It may be, for example, that initial, relatively automatic processes are intact but that subsequent, higher-order stages are disrupted, shedding light on the neural and psychological mechanisms involved in the emotion dysregulation seen clinically. In the present study, the impact of childhood adversity on cortical activity prompted by emotional stimuli and by down-regulation instructions was probed by comparing those participants for whom a structured interview indicated a high stress load during childhood with those who had not experienced substantial adversity.

Three hypotheses were examined. (1) If the input-oriented aspects of emotion regulation imply perceptual appraisal, an early modulation of event-related activity should be expected [27–30]. (2) If the ability to control the emotional response by cognitive reappraisal was undermined by affective disorder and/or childhood experiences, modulation of brain activity at later intervals after stimulus onset was expected to vary with the instruction to down-regulate the emotional response to pictures, differentially in patients versus healthy participants and in patients with highly stressful versus normal childhood histories. (3) Early life stress will moderate such relationships.

## 2. Materials and Methods

### 2.1. Participants

Twenty-seven inpatients with MDD, 15 with BPD, and 28 participants without psychological diagnoses volunteered to participate (see Table 1 for demographic and clinical information). Patients were recruited from the local Center for Psychiatry and identified by treating psychiatrists as meeting an International Classification of Diseases 10th Revision (ICD-10) diagnosis of BPD (F60.31) or MDD (F31–33) as the primary diagnosis. Seven patients met criteria of both diagnoses. (Given that F60.31 was the primary ICD diagnoses in these patients, they were assigned to the BPD group in Table 1 and in the analyses.) Patients were in a stable, remitted state allowing MEG data collection and the stress-history interview. They were included if they had never suffered from any neurological condition including head injury with loss of consciousness. As the center primarily serves long-term patients, most were medicated. Healthy participants were selected to be comparable to the patient sample with respect to age and gender. They were included if they did not meet criteria for a psychological disorder (screened by the M.I.N.I. interview, [31]), had never taken psychoactive medication, and had never suffered from any neurological condition including head injury with loss of consciousness. As evident from Table 1 the patient and the healthy control (HC) groups did not differ with respect to age and gender, though patients had less education. MDD patients were older than the other groups, and BPD patients were almost entirely women. These group differences are common in representative samples. Severity of depressive symptoms was assessed with the German version of Beck Depression Inventory II [33]. As shown in Table 1, diagnostic groups did not differ in depression scores, and both groups scored higher on depression than HC.

### 2.2. Stimuli and Procedures

The protocol was approved by the ethics committee of the University of Konstanz. Prior to the study, participants were informed in detail about the procedures and provided written informed consent. Then they were instructed about strategies of emotion downregulation by reappraisal. (The following instruction about the experiment was given to the participants. In the following you will see neutral and unpleasant pictures, which you will appear for about 2 sec. Before every picture you will see a black screen with a cross in the middle for about 2 sec as well. This cross could be white or blue. The white cross signals you just to watch the picture that follows. This picture could be either a neutral or an unpleasant one. After the blue cross there will show up an unpleasant picture. Unpleasant picture can make you feel stressed or uneasy. To diminish this feeling you should try one of the following strategies. You can prepare yourself by thinking that the scene in the unpleasant picture is not real or from a movie. Or you can try to think the scene in the unpleasant picture will have a positive ending. Like when you see an injured person on the picture you can think that the person will get some help and will be fine. But what you should not do is to think of some unrelated thoughts that will lighten up your emotions like “Today is a nice day,” because you should work with the contents of the pictures. Which of the strategies do you think will work best for you? The blue cross signals you to perform the strategy that works better for you to down-regulate your emotions. Just to make it easier for you we let you know what pictures you will see. The neutral pictures show some normal looking faces and household objects. The unpleasant pictures show harassment by animals or humans and mutilations.). During practice trials sample pictures (not presented during subsequent MEG recording) were presented with the instruction to verbalize the selected strategy to down-regulate the response to the unpleasant pictures. If necessary, subjects were coached on strategy. As a manipulation check participants were asked to report after the experiment what strategies they had used for downregulation. Participants received a bonus of 50 € for completing the experimental session and the interview (see below), which altogether lasted 3-4 hours.

The stimulus set comprised 30 unpleasant, high-arousing and 30 neutral, low-arousing color images taken from the International Affective Picture System (IAPS; [34]). (The numbers of the IAPS pictures used were the following: unpleasant (1050, 1090, 1110, 1113, 1120, 1201, 1220, 1300, 1301, 1930, 3019, 3061, 3150, 3160, 3181, 3213, 3400, 6230, 6243, 6260, 6313, 6350, 6360, 6540, 6560, 6570, 6821, 7361, 8230, 9321) and neutral (2190, 2200, 2210, 2230, 2570, 2840, 5500, 7000, 7002, 7009, 7010, 7020, 7025, 7035, 7050, 7080, 7100, 7150, 7160, 7170, 7175, 7190, 7217, 7224, 7233, 7235, 7550, 7700, 7950, 9070). Unpleasant pictures included harassment by animals or humans (similar to [19]) and mutilation (selection approved by the collaborating psychiatrists at the Center for Psychiatry). Neutral pictures were similar to the design of Moser and colleagues [19] and show neutral faces and household objects.) Unpleasant and neutral pictures differed in IAPS normatively rated valence (M ± SD unpleasant: 2.85 ± 0.66, neutral: 4.93 ± 0.27) and arousal (M ± SD unpleasant 6.29 ± 0,62, neutral: 2.83 ± 0.57). For the two Instruction conditions, passive viewing and down-regulation, the same unpleasant pictures were used but presented in different order. Physical picture parameters such as brightness, contrast, distribution of color, and complexity did not differ for the two Emotion categories (neutral and unpleasant). Pictures were presented via a projection system on a screen about 50 cm from the subject's eyes. Across 180 trials, pictures were pseudorandomly presented in two series of 90 trials. Each series lasted about 10 min and was separated by a 1 min break (black screen). As in typical EEG studies using this emotion-regulation paradigm [19, 22, 35], conditions were restricted to downregulation of emotion to unpleasant pictures, but not upregulation or pleasant pictures, in order to limit duration of measurement.

Participants were asked to fix their gaze on the middle of the screen and to avoid eye and head movements. Each trial started with the presentation of a white or blue cross, which appeared in the center of the screen for 2000 ms and served as a cue for picture emotion type and instruction. At cross offset, a picture appeared for 2000 ms. Trials were separated by picture offset to cross-onset intervals of 2000–2500 ms. The white cross was a signal that a neutral or unpleasant picture would be presented at the offset of the cross and that participants were to view it passively. The blue cross signaled that an unpleasant picture was coming, during which participants were instructed to implement the previously trained mental strategy to down-regulate their emotional response to the picture. Thus, 30 watch-neutral trials, 30 watch-unpleasant trials, and 30 down-regulate-unpleasant trials were presented, in pseudorandom order.

Childhood adverse experiences were assessed in an interview on a different day than the MEG session. The interview used the German version of the Early Trauma Inventory (ETI [32, 36]). Stress load was defined as the number of reported events summed across four domains (emotional abuse/neglect, physical abuse/punishment, general trauma, sexual abuse) between preschool age and early adolescence (age 3–16, as per [37]). BPD and MDD groups did not differ in total stress load (see Table 1). In addition, participants filled out the German version of the Emotion Regulation Questionnaire (ERQ, [38]). Scores on the reappraisal (ERQr) and the suppression (ERQs) subscales served as trait measures of emotion regulation style.

### 2.3. MEG Recording, Data Reduction, and Analysis

High-resolution MEG and nonparametric cluster-based analyses were used. Event-related magnetic fields (ERFs) in scalp sensor space served to examine the temporal sequence of perception-regulation processes as described in [19]. MEG was recorded with a 148-sensor magnetometer (MAGNES 2500 WH, 4D Neuroimaging, San Diego, USA) in a magnetically shielded room while participants were in a supine position. Prior to each measurement, the participant's nasion, inion, Cz, left and right ear canal, and head shape were digitized with a Polhemus 3Space Fasttrack. Neuromagnetic data were continuously recorded in DC mode with a sampling rate of 678.17 Hz. Data were analyzed using the MATLAB-based toolbox *Fieldtrip *[39]. Independent component analysis (ICA, [40]) was used to partial out heart and eye-blink artifacts. Epochs containing movement artifacts and channel jumps were rejected based on visual inspection. The number of artifact-free trials entering analysis did not differ by group or condition (mean±standard deviation for HC watch-unpleasant condition, 55.2 ± 4, watch-neutral condition, 55.1 ± 4.2, down-regulate condition, 55.1 ± 3.7; for MDD patients watch-unpleasant condition, 56.0 ± 2.5, watch-neutral condition, 55.3 ± 3.3, down-regulate condition 54.7 ± 3.3; for BPD patients watch-unpleasant condition, 56.8 ± 2.6, watch-neutral condition, 56.6 ± 2.3, down-regulate condition, 56.7 ± 2.9). Data were low-pass-filtered using a two-pass Butterworth filter with a filter order of 6 and a low-pass cutoff of 40 Hz. Event-related fields (ERFs) were baseline-adjusted using 2000 ms before cross onset. Of interest were responses to cross onset and responses to picture onset. Analysis of the former served to evaluate group differences in the processing of instruction signals. Analysis of the latter served to evaluate group differences in the processing of picture content (emotion effect) as well as instruction (regulation effect) and was of primary interest.

**Figure 1:**
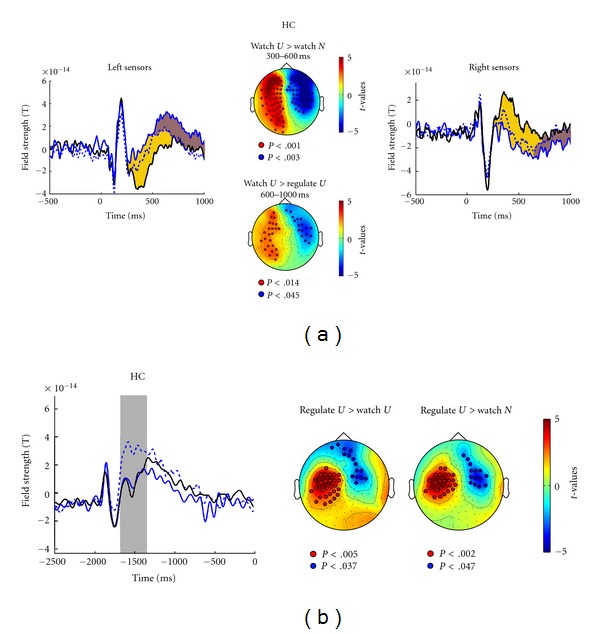
(a) Time course of field strength around picture onset at 0 ms for watch-neutral (solid black lines), watch-unpleasant (solid blue lines), and down-regulate-unpleasant conditions (dotted blue lines) for healthy control participants (HC). The left panel illustrates left-hemisphere sensors, the right panel right-hemisphere sensors. In- and outgoing magnetic fields explain the opposite direction of the effect. Periods during which conditions differ significantly are marked in yellow for watch-unpleasant versus watch-neutral (Emotion effect) and in brown for watch-unpleasant versus down-regulate-unpleasant (Instruction effect). The center panel presents *t*-maps projected onto a schematic top view (left = left) testing watch-unpleasant versus watch-neutral (Emotion effect) 300–600 ms after picture onset (top) and comparing watch-unpleasant versus down-regulate-unpleasant (Instruction effect) 600–1000 ms after picture onset (bottom). Sensors defining the significant cluster are marked by open circles). (b) Time course of field strength preceding cross cue onset (−2000 ms) to picture onset (0) ms for watch-neutral (solid black line), watch-unpleasant (solid blue line), and down-regulate-unpleasant (dotted blue line) conditions for HC group. The gray bar marks the epoch of significant differences between conditions. *t*-maps projected onto a schematic top view are plotted below, testing down-regulate-unpleasant versus watch-unpleasant conditions and testing down-regulate-unpleasant versus watch-neutral. Red and blue colors represent sensor clusters that show significant (*P* < .01) differences in field strength between conditions. Sensors defining the significant clusters are marked by open circles. The different direction of effects explains positive and negative *t*-values referring to the left (positive *t*-values, red) and right (negative *t*-values, blue) hemisphere ERF.

Data analyses were based on the time course of ERFs from 500 ms before to 1000 ms after picture onset for the 28 healthy control subjects (HC, see Figure 1(a)). Condition effects are evident 250–1000 ms after picture onset. Time windows in which ERFs varied with emotional stimulus processing and emotion regulation instruction were determined using nonparametric cluster-based permutation *t*-tests that control for type I error rate in the context of multiple comparisons across multiple sensors and many subjects [41]. This procedure identified two time windows with significant differences between conditions, 300–600 ms and 600–1000 ms after picture onset. For these intervals, the initial analysis on the 28 HC identified clusters of sensors. Sensor clusters were identified as differentially active when differences between conditions exceeded a threshold of significance at the 5% level. Via 5000 random permutations of the data, the cluster-level statistic was defined as the sum of *t*-values within each cluster containing at least 5 adjacent sensors. The obtained *P* values index the null-hypothesis probability (no difference between conditions) of observing a maximum (minimum) cluster-level statistic larger (smaller) than the observed cluster-level statistics. This procedure assessed the surface distribution of significant ERF differences between conditions for the respective time windows across multiple sensors. Results of the same analyses for the time window of cross cue processing are presented in Figure 1(b), with separate averages for the three conditions (watch-unpleasant, watch-neutral, down-regulate) reflecting responses for the white cross (signaling watch-unpleasant and watch-neutral) and for the blue cross (down-regulate).

After cluster identification, group and condition effects were statistically evaluated at a sensor in the middle of the significant left-hemisphere sensor cluster having the maximum field strength in each participant. A sensor in the middle of the cluster was chosen to avoid effects of unrelated neighboring activity. Resulting scores were evaluated in separate ANOVAs with the orthogonal between-subject factors Group (patients versus HC) or Diagnosis (MDD versus BPD) and the within-subject factors Emotion (watch-neutral versus watch-unpleasant) or Instruction (watch-unpleasant versus down-regulate-unpleasant).

An additional ANOVA evaluated the impact of childhood adversity in patients with the between-subjects factor Stress group. Assignment to the high-stress or low-stress group was based on the total number of adverse events experienced across age 3–16 as reported in the interview. Those in the upper 30% of the distribution (*N* = 15) were assigned to the high-stress group, and those in lower 30% (*N* = 15) were assigned to the low-stress group. (This ANOVA was confined to patients, as all HC reported low stress load.) Moreover, as BPD and MDD did not differ with respect to stress load (see Table 1), diagnosis was not a factor in this ANOVA. High stress load was not particularly characteristic for patients with comorbid MDD and BPD (4 were assigned to the low-stress, 3 to the high-stress group). Relationships between ERF scores, emotion regulation (ERQ) style, and depressive symptoms were assessed by Pearson correlations.

## 3. Results

### 3.1. Event-Related Fields in Healthy Participants

An initial manipulation check demonstrated findings in the present MEG data set similar to those in EEG ERP studies.  Figure 1 shows that the Emotion and Instruction manipulations affected brain responses at different latencies. Unpleasant pictures evoked a stronger response than did neutral pictures 300–600 ms after picture onset. Instructions to down-regulate eliminated that difference 600–1000 ms into the picture. The ANOVA comparing watch-neutral and watch-unpleasant conditions verified the Emotion effect at 300–600 ms (*F*(1,27) = 39.21, *P* < .01). An analysis comparing all three conditions (watch-neutral, watch-unpleasant, down-regulate-unpleasant, *F*(2,54) = 30.88, *P* < .01) and post hoc paired *t*-tests confirmed weaker response during the down-regulate-unpleasant condition than the watch-unpleasant condition (*t*(27) = −2.31, *P* < .05), which in turn was weaker than the watch-neutral condition (*t*(27) = −6.13, *P* < .01).

Unpleasant pictures prompted a different pattern of results later in the trial. Figure 1(a) shows that 600–1000 ms into picture presentation the ERF during the down-regulate-unpleasant trials was more similar to that during watch-neutral trials than to that during watch-unpleasant trials, suggesting suppressed activity with down-regulation instructions. The condition effect for the 600–1000 ms window (*F*(2,56) = 8.68, *P* < .01) and post hoc *t*-tests confirmed the difference between watch-unpleasant and down-regulate-unpleasant conditions (*t*(28) = 3.05, *P* < .01), while there was no difference between watch-neutral and down-regulate-unpleasant conditions.

As shown for HC in Figure 1(b), ERFs differed around 300 ms after the onset of the cross cue. The blue cross signaling later downregulation evoked a larger response than the white cross signaling passive viewing. This effect mirrors the effect of rare versus frequent stimuli in the latency range of the P300 event-related potential of the EEG. Permutation *t*-tests verified this effect for a left-hemisphere central-posterior sensor group. No differential ERF was obvious prior to picture onset (in the time window of stimulus-preceding negativity).

### 3.2. Neuromagnetic Correlates of Emotion Processing and Regulation in Patients

**Figure 2:**
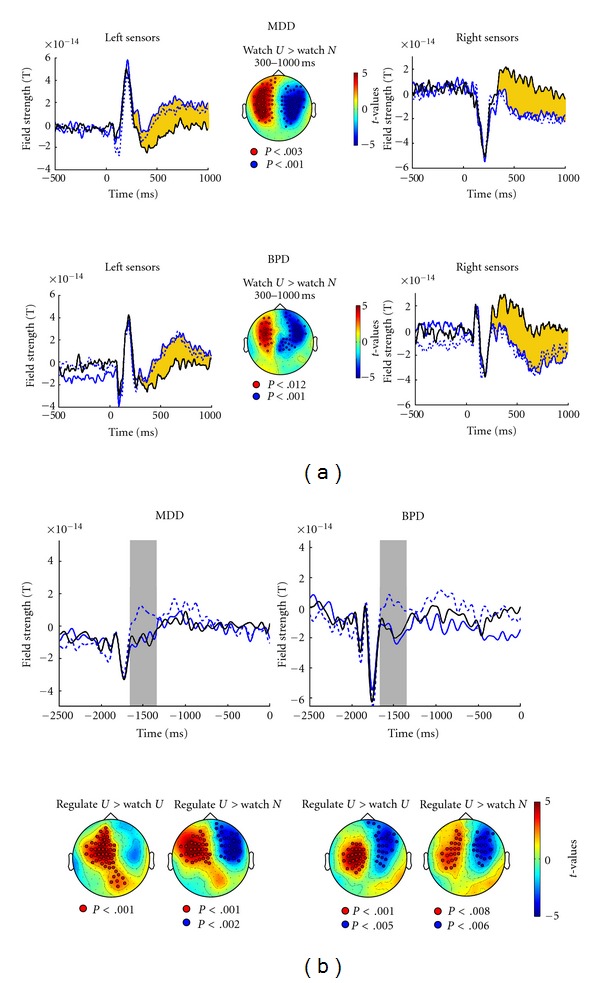
(a) Time course of field strength around picture onset at 0 ms and sensor clusters of statistical differences between conditions defining the Emotion effect for MDD patients (top) and BPD patients (bottom). Line types and so forth as in Figure 1(a). (b) Time course of field strength preceding picture onset at 0 ms, with cross cue onset at −2000 ms, and sensor clusters of statistical differences between conditions for MDD (left) and BPD (right) patients. Line types, gray bars, and so forth as in Figure 1(b).

With present MEG data essentially replicating an earlier emotion effect and a later instruction effect [19], the next question was how MDD or BPD patients might differ.  Figure 2(a) illustrates the time course of ERF to picture onset for the three conditions (watch-neutral, watch-unpleasant, down-regulate-unpleasant) in patients. Differential responses to neutral and unpleasant pictures during passive viewing were sustained for much of the interval (300–1000 ms), whereas down-regulate instructions had no effect in patients. *t*-statistics confirm differences between watch-unpleasant and watch-neutral and between down-regulate-unpleasant and watch-neutral between 300–1000 ms at bilateral frontotemporal sensors. The ANOVA comparing groups and conditions for the selected sensor confirmed the Emotion effect at 300–600 ms (*F*(2,67) = 75.03, *P* < .01). At 600–1000 ms after picture onset, HC showed ERF modulation when instructed to down-regulate, but patients did not. This lack of an Instruction effect was reflected in a Group × Condition interaction for the watch-unpleasant and down-regulate-unpleasant conditions (*F*(2,136) = 4.79, *P* < .01) and for all three conditions (*F*(4,134) = 3.58, *P* < .05, GG *ε* =  .95). MDD and BPD ERFs did not differ, nor did the seven comorbid patients differ from those with a single diagnosis of either MDD or BPD (*F* = 1).

**Figure 3:**
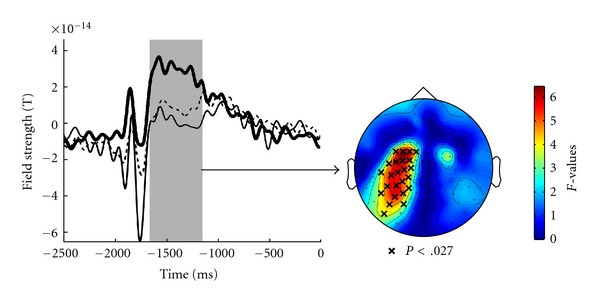
Time course of field strength as in Figure 2(b) for the down regulate condition (blue cross cue) averaged separately for HC (solid thick line), MDD patients (dashed line), and BPD patients (solid thin line). The gray bar marks the epoch of significant group differences. The right panel illustrates sensors defining the significant cluster marked by “x” (referring to the group differences).

Responses to cross cues were smaller in patients than in HC (Figure 2(b)). However, similar to HC, patients exhibited larger responses to the (less frequent) blue cross signaling emotion regulation than to the (more frequent) white cross signaling passive viewing. *t*-statistics confirm these effects, whereas Figure 3 illustrates the smaller activation by rare stimuli in patients (main effect group) between 300 and 700 ms post-cue-onset at left-hemisphere central-posterior sensors. No group or condition differences were evident for the remaining anticipatory interval preceding picture onset. In summary, affective disorder was associated with disrupted emotion regulation and not with initial processing of emotional stimuli, supporting hypothesis 2 rather than hypothesis 1.

### 3.3. Emotion Regulation as a Function of Childhood Stress

**Figure 4:**
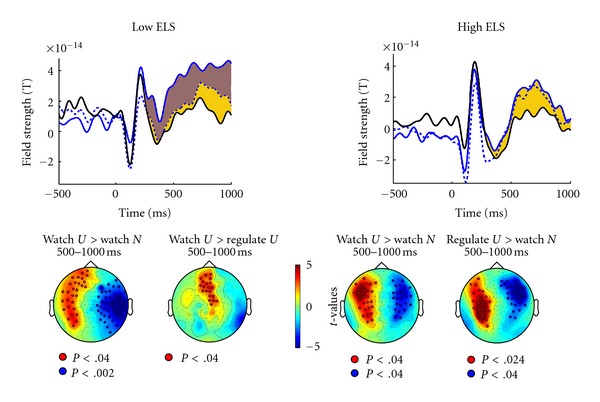
Top: time course of field strength around picture onset at 0 ms (as in Figures 1(a) and 2(a)) for patients with low childhood stress load (*N* = 15, left graph, low ELS = early life stress) and patients with high childhood stress load (*N* = 15, right graph, high ELS). Yellow shading marks epoch of significant Emotion effect. Brown shading marks epoch of significant Instruction effect (present only in low-stress group). Bottom: *t*-maps projected onto schematic top views testing watch-unpleasant versus watch-neutral and testing watch-unpleasant versus down-regulate-unpleasant 500–1000 ms after picture onset. Red and blue colors represent sensor clusters which show significant (*P* < .01) differences in field strength between conditions, with sensors defining the significant clusters marked by open circles (as in Figure 1).

The final hypothesis was about the effect of childhood adversity on emotion processing and/or on instruction to down-regulate. During picture presentation, patients with low stress load exhibited effects of emotion regulation similar to those of HC, whereas patients with high stress load did not (Figure 4). For the low-stress group, differences between watch-unpleasant and down-regulate-unpleasant were evident 600–1000 ms at a frontocentral sensor cluster (Figure 4). Evaluating the impact of stress load on the modulation of ERF by instruction for a sensor with maximum field strength in each patient in the middle of this cluster confirmed that patients with high stress load history failed to show ERP modulation by instructions, whereas in low-stress-history patients the instruction effect was robust (Stress group × Instruction *F*(1,25) = 7.24, *P* = .01; post hoc paired *t*-tests in the low-stress group, *P* < .01). A history of stressful childhood experiences did not modulate ERF prior to picture onset.

### 3.4. Correlational Relationships

Stress load around puberty (age 9–13) varied with BDI depression in MDD (*P* < .05) and with trait emotion regulation as assessed by ERQ (across groups, higher stress load varied with higher scores on the ERQ suppression scale, *r* = .27, *P* < .05, and with lower scores on the ERQ reappraisal scale, *r* = −.28, *P* < .05). Trait emotion regulation, which was reportedly poorer in patients than in HC (see Table 1), varied with BDI depression mainly in BPD, with higher depression scores varying with less use of reappraisal, *r* = −.65, *P* < .05, and with higher tendency for suppression, *r* = .50, *P* = .05 (for the entire sample, *r* = −.41, *P* < .01, and *r* = .33, *P* < .05). In HC, a larger instruction effect on ERF tended to vary with reappraisal (ERQr) (*r* = .35, *P* = .07). In MDD, this instruction effect was related to lower BDI depression (*r* = −.34, *P* = .09). No relationships were found for early processing of the emotional stimuli.

## 4. Discussion

 The present neuromagnetic results provided support for hypothesis 2 (over hypothesis 1) and for hypothesis 3. Results confirmed a normal sequence of event-related brain responses modulated by experimental variables [19–22, 35]. Thus, processing of the emotional content begins to unfold before regulation strategies dampen the response. This is in line with models of emotion regulation that define and distinguish perceptual input-oriented processes of monitoring, appraisal, or evaluation of an emotional stimulus and response- or output-oriented regulation processes that may include cognitive reappraisal or response modulation [9–12]. The early ERF modulation by stimulus valence (300–600 ms) may correspond to the electrophysiological event-related potential component EPN (early posterior negativity), which has been associated with initial selective attention and automatic discrimination of salient stimuli or stimulus valence [26–30]. The subsequent ERF modulation by instruction to down-regulate may correspond to the late positive potential (LPP), which varied with emotion regulation in [19–21] and which has been related to memory-based emotion evaluation [30, 42, 43]. Sources of LPP have been confirmed for posterior (visual) areas when subjects process emotional (IAPS) pictures [43] and also for regions related to emotion processing (insula, cingulated gyrus) and premotor areas [44]. Thus, an anterior distribution as in the present study is in line with other findings.

Differential ERFs in response to the cross cue stimuli did not suggest any abnormality associated with affective disorder. These differential responses were related to stimulus frequency rather than to stimulus meaning. Larger EEG ERP amplitude to rare than to frequent stimuli is a common finding, known as the “oddball” effect (e.g., [45]).

 Fronto-temporal neuromagnetic activity distinguished responses to unpleasant and neutral stimuli similarly across groups. However, patients did not show normal activity suppression when instructed to down-regulate emotion to unpleasant stimuli. Thus, present results suggest a specific impairment of emotion regulation in patients with affective disorders. Hemodynamic imaging studies have related dysfunctional emotion regulation to reduced prefrontal activity. In the present MEG data, this was presumably manifest in the lack of differential activity at frontotemporal sensor clusters. This conclusion is in line with reports of temporal hypofunction in depression [46]. However, early ERFs distinguishing responses to unpleasant and neutral stimuli suggest that perceptual appraisal of emotional stimuli was not impaired in patients with affective disorders. Previous MEG studies showed similarly intact modulation by arousal (difference between neutral and pleasant/unpleasant pictures) despite reduced early activity to emotional pictures [25]. Present results of a slightly later time window of modulation by arousal (starting at 300 ms) and overall activity similar to that of HC may result from differences in the experimental designs: modulation may occur later when more processing time is allowed [21, 35]. Moreover, effects of passive viewing may be different when combined with a regulation task.

Deficient emotion regulation in patients may have resulted from generally reduced cognitive processing efficiency, hence, less efficient implementation of cognitive reappraisal strategies. MDD patients, who have been reported to suffer from cognitive deficits, may have been particularly impaired in implementing instructions for down-regulation by cognitive reappraisal. Indeed, patients not only failed to show ERF differentiation under down-regulation instruction but showed smaller responses to the signal instructing downregulation. Group differences were prominent in left-hemisphere, central-posterior regions that may be associated with the translation of the nonverbal signal (cross) into a working-memory-based verbal representation of the previously learned strategies of reappraisal. This result might suggest a failure to adequately process the instruction and, as a consequence, implement the down-regulation instruction properly, while the automatic differential response to arousing pictures was unaffected by this cognitive deficit. However, as patients exhibited similarly significant differences between the different cross cue stimuli, a general failure to cognitively process signal stimuli seems unlikely. Moreover, the manipulation check and the emotion regulation by instruction in patients without history of childhood adversity argue against general cognitive deficits explaining away the lack of regulation effect. Finally, cognitive dysfunction constitutes a prominent symptom in major depression, whereas disturbed executive function of a different sort is primarily reported in BPD [47]. In the present study, BPD and MDD patients showed similarly reduced “oddball” responses to the signal stimuli. Thus, for several reasons, a general confound seems unlikely.

The present assessment of emotion regulation was restricted to the down-regulation instruction and to unpleasant stimuli. In healthy subjects, similar effects of regulation instructions have been reported for pleasant stimuli [22] and for the instruction to increase emotional response. A comparison of conditions might elucidate a particular sensitivity for negative stimuli [48, 49] (and, hence, difficulty in down-regulating emotional responses) in MDD relative to a general inability to implement regulation instructions. As in other studies [19, 22, 35]), restricting conditions to downregulation and unpleasant stimuli in the present study balanced the requirements of trial repetition in EEG/MEG-measurement against duration of measurement. However, follow-up studies with complementary conditions would be useful in the evaluation of emotion regulation in patients.

 Marked experience of childhood adversity modified emotion regulation, supporting hypothesis 3. Without such experiences, normal emotion regulation occurred to some extent in patients with affective disorders. An impact of early life stress on emotion processing has been proposed and explained by stress-mediated alterations of reward processing and motivation states [50, 51], but interactions with other pre- and postnatal influences have to be assumed that add to interindividual variability. Childhood adversity is supposed to affect the normal development of brain and neuroendocrine systems related to stress and affect processing [37, 50]. Future studies could focus on the interaction of disease-specific factors and childhood experiences in their impact on emotion regulation and on brain mechanisms potentially involved in such processes. Still, present results suggest that emotion regulation style is influenced by childhood experiences and by current symptom severity, both exerting some influence on the ability to implement emotion regulation in an experimental task.

To conclude, dysfunctional emotion regulation has been discussed for affective disorders without specifying whether emotional input processing and/or response regulation are primarily affected. For patients with MDD and BPD the present study confirmed deficient instructed downregulation of responses to unpleasant pictures in an experimental task, whereas the differential processing of unpleasant relative to neutral stimuli proved to be unimpaired. Although causal links cannot be inferred from the present results, they suggest that adverse childhood experiences influence emotion regulation.

## Figures and Tables

**Table 1:** Demographic and clinical information for groups.

	Healthy control (HC, *n* = 28)	Major depressive disorder (MDD, *n* = 27)	Borderline personality disorder (BPD, *n* = 15)	Group effects
Gender (m/f)	13/15	16/11	1/14	Patient-HC: n.s.
				MDD-BPD: Chi2(2) = 11.1**

Age (M ± SD)	31.0 ± 12.5	40.8 ± 12.7	26.1 ± 6.4	Patients-HC: n.s.
				HC and BPD < MDD** *F*(2,67) = 9.08**

Years of education	11.8 ± 1.5	10.3 ± 1.6	9.7 ± 1.1	Patients-HC: *t*(68) = 4.52, *P* < .01
(M ± SD)				MDD-BPD n.s.

BDI (M ± SD)	3.3 ± 3.0	21.4 ± 12.3	29.4 ± 15.5	Patients-HC: *F*(1,68) = 75.00**
				MDD-BPD: n.s., MDD and BPD > HC: *F*(2,67) = 39.15**

ERQr (M ± SD)	4.8 ± 0.9	3.8 ± 1.1	3.7 ± 1.3	Patients-HC: *F*(1,68) = 13.74**
ERQs (M ± SD)	3.3 ± 1.0	4.4 ± 1.3	4.3 ± 1.9	Patients-HC: *F*(1,68) = 10.97** MDD-BPD: n.s.

Stress load (number of events)	14.4 ± 12.5	36.1 ± 26.7	48.7 ± 25.0	BPD-MDD n.s. BPD, MDD > HC** *F*(2,67) = 13.85**
			

*Note*. BDI: Beck Depression Inventory II, German version [33]. ERQ: Emotion Regulation Questionnaire [38]; ERQr: subscale reappraisal subscale; ERQs: subscale suppression subscale. *P < .05, **P < .01, n.s. *P* < .1.
